# Research on Green Total Factor Productivity of Yangtze River Economic Belt Based on Environmental Regulation

**DOI:** 10.3390/ijerph182212242

**Published:** 2021-11-22

**Authors:** Junxia He, Luxia Wang, Decai Tang

**Affiliations:** 1 China Institute of Manufacturing Development, Nanjing University of Information Science & Technology, Nanjing 210044, China; 20211900022@nuist.edu.cn; 2 School of Management Science and Engineering, Nanjing University of Information Science & Technology, Nanjing 210044, China

**Keywords:** Yangtze River economic belt, SBM-undesirable, Malmquist index, green total factor productivity, environmental regulation

## Abstract

With the acceleration of industrialization and urbanization, the Yangtze River Economic Belt (YREB) is facing many environmental problems that need to be solved in the process of development. This paper aims to analyze the environmental governance effects of nine provinces and two municipalities in the Yangtze River Economic Belt from 2009 to 2018. Firstly, based on the input-output index, the slacks-based measure (SBM) undesirable model and Malmquist (ML) index were used to measure the green total factor productivity (GTFP) of the YREB from 2009 to 2018. The results showed that the technological progress index contributed the most to the GTFP of the YREB, followed by the pure technical efficiency index and the scale efficiency index. Environmental regulation has no significant impact on the GTFP of the YREB. Secondly, by analyzing the effect of environmental governance in the YREB, the results show that the main reasons for the ineffective environmental governance in the YREB are the redundant input of environmental resources, excessive unwanted output, and low harmless treatment rate of municipal solid waste, rather than the low level of urban environmental management. Finally, this paper provides recommendations for the ineffective provinces and municipalities of the YREB to further optimize the input-output factors of environmental governance.

## 1. Introduction

**Figure 1:**
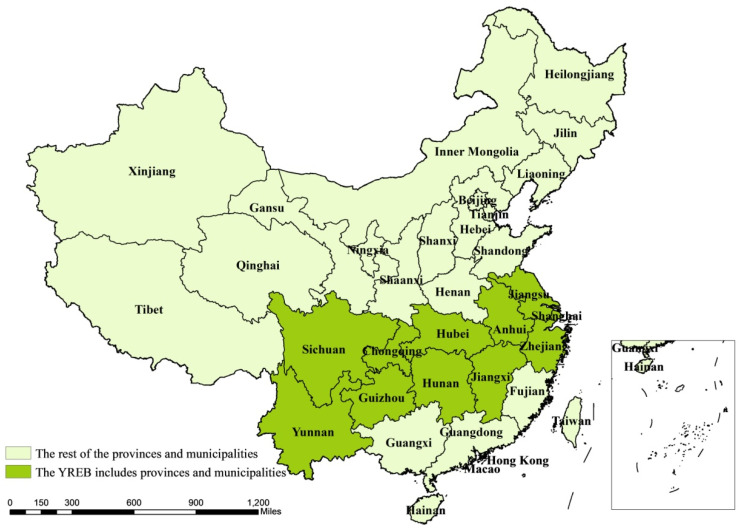
The Yangtze River economic belt research area (dark green).

The Yangtze River Economic Belt (YREB) comprises nine provinces (Anhui, Guizhou, Hubei, Hunan, Jiangsu, Jiangxi, Sichuan, Yunnan, and Zhejiang) and two municipalities (Chongqing and Shanghai) and sits on a total land area of over two million square kilometers, and accounts for approximately 40% of China’s GDP (Figure 1). Since the reform and opening up, the comprehensive strength of the YREB has been in a leading position in the regional economic belt, and its role of strategic support for China’s economy has become increasingly prominent. In April 2018, Chinese president Xi Jinping pointed out at the Symposium on Deepening the Development of the YREB that the YREB should explore new ways to promote ecological priority and green development in a coordinated way and should be built into an integrated and efficient economy. Green development, as an important part of the high-quality development of the YREB, should be promoted in a coordinated way by efficiency reform and power reform [1]. However, in the process of the development of the YREB, the green industrial transformation and upgrading are slow, and the imbalance of regional development has been emerging. Consequently, strict environmental regulation and technological innovation are the only way for the Yangtze River economy to achieve balanced development of economic development and environmental protection [2].

At present, the YREB is in a critical period of industrial restructuring, transformation and upgrading, and is in urgent need of transforming from an extensive economy to one that is supported by productivity. On the one hand, in the context of the “green” revolution sweeping the world, accelerating the “green” productivity improvement and clean technology innovation has become an important approach to enhance green development. On the other hand, the environmental carrying capacity of the YREB is very limited, and the economic development mode with high energy consumption, high pollution, and high emissions cause the continuous deterioration of the environmental situation. As a result, the Chinese government has to implement strict environmental regulation policies to curb environmental deterioration [3]. What influences will environmental regulation have on the economic development of the YREB? How is the effect of environmental governance in the YREB? From the perspective of environmental regulation, considering the undesirable output, this paper calculates the environmental green total factor productivity of the YREB and analyzes its influencing factors, aiming to answer the above questions. By analyzing and answering the above questions, this paper can not only provide research insights for scholars focusing on the regional economy to investigate the economic growth from a more comprehensive perspective by taking undesirable outputs into account when calculating green total factor productivity, but also raise concerns of other economic regions in China such as Pearl River Delta and Beijing-Tianjin-Hebei to lead a green and effective development by identifying the driving force of GTFP.

The rest of this paper is structured as follows. Section 2 provides a literature review and research hypothesis. Section 3 introduces the measurement method of green total factor productivity, data sources, and variables. Section 4 calculates the green total factor productivity and analyzes its influencing factors. The environmental governance of the YREB is also studied in this part. Section 5 provides the conclusions and suggestions of this paper.

## 2. Literature Review and Research Hypothesis

### 2.1. Literature Review

With increasingly severe resource and environmental constraints, the major concern of economic development has become the reduction in industrial pollutant emissions and the promotion of green industrial development. The fundamental change of China’s industrial development mode is to comprehensively improve the green total factor productivity of industrial development [4]. Green total factor productivity is an indicator to measure the quality of economic growth. It is a concept derived from the total factor productivity, which, beyond the traditional GDP, is used to reflect the impact of technological progress and efficiency improvement during the process of economic growth [5,6]. By incorporating environmental variables into the production framework, GTFP comprehensively considers socio-economic as well as resource and environmental factors, thus has become more and more popular among scholars and is widely employed to calculate the growth quality at country, region, and industry levels [7]. Furthermore, GTFP can be further decomposed into technical efficiency and technological progress, the former reflects to what degree the current technology employs the resources and the latter reflects the impacts from out factors on the decision-making units.

As for the measurement method of green total factor productivity, data envelopment analysis (DEA) was first proposed by Charnes, Cooper and Rhodes in 1978 [8]. Involving multiple fields such as mathematics, economics, and operations research, DEA is a non-parametric analysis method based on the comparison between evaluated objects and was widely applied by scholars in the late 20th century. This technique computes efficiency by linear programming within two steps: constructs the frontier from the data and computes the distance of each unit to the frontier [9]. Subsequently, Tone and Fare [10,11,12] created and developed SBM directional distance function on the basis of previous studies, which provided a new approach for scholars to perform further study. In the context of the carbon cycle in the YREB, Liu et al. [13] calculated China’s agricultural green total factor productivity based on carbon emissions and studied its dynamic evolution trend with a panel data model by using the kernel density estimation method. The influencing factors of China’s agricultural green total factor productivity were also investigated in their study. Liu et al. [4] used the SBM model to calculate the green production efficiency of China’s industry, and the results showed that the green production efficiency of China’s industry was generally at a low level and was seriously polarized. Li et al. [14] believed that the growth of green total factor productivity in the manufacturing industry mainly depends on technological progress rather than technological efficiency. As environmental issues in the YREB have been paid more and more attention, Wang et al. [15] studied the change of green total factor productivity from the perspective of green technological innovation. Tian et al. [16] suggested that green innovation efficiency has a significant positive spatial correlation. Gou et al. [17] studied the changes of environmental total factor productivity in the YREB from the perspective of regional green development. Hou et al. [18] demonstrated that market integration can promote the improvement of green total factor productivity. Song et al. [19] used enterprise-level data to investigate the impact of OFDI on GTFP under economic uncertainty.

While focusing on the change of green total factor productivity, scholars also explored the impact of environmental regulation on green total factor productivity and the effect of environmental governance from various perspectives. Based on the network DEA method, Wang et al. [20] calculated overall environmental regulation efficiency in five Chinese urban agglomerations (Beijing-Tianjin-Hebei, Chengdu-Chongqing, Middle Reaches of Yangtze River, Pearl River Delta, and Yangtze River Delta) from 2000–2014 and found that the Yangtze River Delta (YRD) and Beijing-Tianjin-Hebei region had the highest environmental regulation efficiency, meanwhile, the Pearl River Delta region was the most effective in environmental governance. Furthermore, the rest of the region was at a low level both on environmental regulation efficiency and environmental governance. Liu et al. [21] believed that the overall environmental regulation efficiency of the YREB is in a weak effective state. Specifically, the resource gathering in the economically developed area is in favor of the improvement of environmental regulation efficiency, while the environmental regulation in the economically disadvantaged area is more likely to be invalid due to the lack of resources. When it comes to the central cities, the environmental regulation efficiency is high and the ecological efficiency difference between city clusters is significant. Chen et al. [22] found that formal environmental regulation has a promoting effect on green total factor productivity, but Su and Zhang [23] believed that the impact of environmental regulation on green economic efficiency presents a U-shaped curve, and such relationship is more obvious at the eastern and national levels compared with the western region. Cao et al. [24] verified the inverted U-shaped relationship between environmental regulation and economic growth by taking the YRD region as a sample. Based on the SBM model and ML index and from the perspective of input-output optimization, Tang and Bethel [25] found that the efficiency of environmental regulation in the YREB was not only low but also deteriorated from 2003 to 2013. Chen et al. [26] identified that the overall efficiency of environmental regulation in the YREB showed a declining trend from 2003 to 2014, with regional differences being significant. It was also suggested that management and scale optimization levels were the main factors hindering the growth of total factor productivity. Peng et al. [27] found that from 2012 to 2016, the overall environmental governance is improved, but from the dynamic point of view, the air pollution generated in the process of urbanization hurt the urban environmental governance performance. Moreover, environmental regulation efficiency of different provinces and municipalities remain consistent with the regional economic condition and comprehensive strength.

Based on the above observation, scholars mainly adopt the SBM model and ML index to measure green total factor productivity by taking into account the undesired output. Meanwhile, green total factor productivity is decomposed into technical progress and technical efficiency for analysis. It is also believed that the impact of environmental regulation on green total factor productivity presents a U-shaped relationship, but there are obvious regional differences in the effect of environmental regulation. With the improvement of the efficiency of environmental regulation, the environmental governance of provinces and municipalities get promoted as well. This paper investigates the change of green total factor productivity and environmental governance in the YREB considering environmental regulation. Compared with the literature, the main contributions of this paper are as follows. Firstly, considering the influence of undesired output on green total factor productivity, based on the actual situation of industrial development in the YREB, the change of green total factor productivity in the YREB, which is decomposed into technological progress and technological efficiency, is accurately measured and analyzed. Secondly, the impact of environmental regulation on green total factor productivity is considered and empirically studied. Thirdly, the reasons for the ineffectiveness of environmental governance are analyzed, based on which the corresponding suggestions are put forward to solve the existing problems of the YREB.

### 2.2. Research Hypotheses

With respect to the impact of environmental regulation on the GTFP, there are totally different effects [28]. On the one hand, the implementation of environmental regulation would force local enterprises to pay taxes for pollution, due to which enterprises will purchase environmental protection equipment or reduce their environmental pollution by investing in advanced technology. To save costs, companies may also choose to expand production scale. That is to say, environmental regulation helps improve the GTFP. On the other hand, enterprises unable to meet the environmental standard will be forced to withdraw from the market because they could not afford to carry out any green technology or equipment. Such a crowding-out effect will harm the GTFP in the long run.

When it comes to the time trend of the green total factor productivity in YREB, the economic situation and political environment play important roles. For example, the outbreak of the financial crisis in 2008 led to a long-term influence on the national economy thus hindering the development of the advanced technology of industries, which in turn limits the increase of the green total factor productivity; the government policy controlling environmental pollution also has potential effects on GTFP. Furthermore, in terms of the contribution of the technological progress and technical efficiency to the green total factor productivity of YREB, the situation of how industrial economy and local environmental regulation policies are coordinated matters since the technological progress and technical efficiency differ from province to province. Specifically, provinces with coordinated development and relatively mature and complete industry distribution have the advantage in employing resources and improving environmentally friendly outputs.

Based on the above discussion, we propose the following hypotheses and test them one by one in Section 4.

**Hypothesis** **1** **(H1).** *GTFP and its decomposition, technological progress, and technical efficiency, are simultaneously influenced by the economic and political environment and differ from province to province, based on the level of the local economic development.*

**Hypothesis** **2** **(H2).** *Environmental regulation has positive impact on the GTFP of YREB, but the impact may be insignificant.*

## 3. Methodology and Data

### 3.1. Methodology

#### 3.1.1. SBM-Undesirable Model

Tone [10,11] first proposed the non-radial SBM model in 2001. Compared with the traditional DEA model, this model can include the desirable and undesirable outputs into the evaluation index system under the consideration of environmental constraints and can include relaxation variables into the objective function. Because the SBM model can effectively solve the measurement errors caused by radial and angle, and the evaluation value of efficiency is relatively accurate, this model is widely used to study the efficiency of environmental governance.

In the SBM-undesirable model, it is assumed that there are *n* decision making units (DMU), and each DMU consists of three parts, namely, *m* kinds of input variables, denoted as $xj=\;\left(x1j,x2j,\ldots ,xmj\right)$, *p* kinds of desirable output variables, denoted as $wj=\;\left(w1j,w2j,\ldots ,wpj\right)$, and *q* kinds of undesirable output variables, denoted as $zj=\;\left(z1j,z2j,\ldots ,zqj\right)$, the model is constructed as follows:

(1) $$\;MIN\rho =\frac{1-\frac{1}{m}\sum_{i=1}^{m}\frac{s_{i}{}^{-}}{x_{i0}}}{1+\frac{1}{p+q}(\sum_{r=1}^{p}\frac{p_{r}{}^{+}}{w_{rj}}+\sum_{t=1}^{q}\frac{z_{t}{}^{-}}{z_{tj}})},$$

(2) $$s.t.\left\{\begin{array}{c} x_{i0}=\sum_{j=1}^{n}x_{ij}\lambda_{j}+s_{i}{}^{-}\;\;i\;=\;1,2,\ldots ,m\\ w_{r0}=\sum_{j=1}^{n}w_{rj}\lambda_{j}-p_{r}{}^{+}\;\;r\;=\;1,2,\ldots ,p\\ z_{t0}=\sum_{j=1}^{n}z_{tj}\lambda_{j}+z_{t}{}^{-}\;\;t\;=\;1,2,\ldots ,q\\ \sum_{j=1}^{n}\lambda_{j}=1\\ \lambda_{j},s_{i}{}^{-},p_{r}{}^{+},z_{t}{}^{-}\geq 0 \end{array}\right.,$$

where $s_{i}{}^{-}$ is the input relaxation variable, $p_{r}{}^{+}\;$is the desirable outputs relaxation variable, $z_{t}{}^{-}\;$is the undesirable outputs relaxation variable, ρ is the efficiency value. When the efficiency value is 1, and $s_{i}{}^{-}=p_{r}{}^{+}\;=z_{t}{}^{-}$, the DMU is effective. Otherwise, when the efficiency value is between 0 and 1, the DMU is ineffective.

#### 3.1.2. Malmquist Index

Considering resource constraints, the undesired outputs can be considered in the analysis of green total factor productivity by using the Malmquist index, so as to understand the impact of undesired outputs on environmental governance [8,23]. Specific functions are as follows:

(3) $${\vec{D}}_{t}\left(a_{t},b_{t},c_{t},d_{t}\right)\;=Max\beta ,$$

(4) $$s.t.\left\{\begin{array}{c} \sum_{i=1}^{I}f^{t}{}_{i}a^{t}{}_{fm}\leq \;\left(1-\beta \right)a^{t}{}_{ih},h\;=1,2,\ldots ,H\\ \sum_{i=1}^{I}f^{t}{}_{i}b^{t}{}_{fm}\geq \;\left(1+\beta \right)b^{t}{}_{im},m=1,2,\ldots ,M\\ \sum_{i=1}^{I}f^{t}{}_{i}c^{t}{}_{fm}=\;\left(1-\beta \right)c^{t}{}_{in},n=1,2,\ldots ,N\\ f^{t}{}_{i}\geq 0,i=1,2,\ldots ,I \end{array}\right.,$$

where $a_{it}$, $b_{it}$ and $c_{it}$ represent the input variable, expected outputs variable and undesirable outputs variable of the ith DMU in the period t, and $f_{i}$ is the return of production scale in the period t. ${\vec{D}}_{t}$ represents the directional distance function under variable return to scale (VRS) with and variable return to scale (VRS) with and without $\sum_{j=1}^{n}\lambda_{j}=1\;$constraints, respectively.

Further, we measure the green total factor productivity by using the Malmquist productivity index, which is defined based on the geometric mean of the two output distance functions, the similar calculation can be found in [6]. Therefore, we can define GTFP as the following formula:

(5) $$\;\mathrm{GTFP}=\sqrt{\frac{1+\vec{D_{t}{}^{i}}\left(a_{t}{}^{i},b_{t}{}^{i},c_{t}{}^{i},d_{t}{}^{i}\right)}{1+\vec{D_{t}{}^{i}}\left(a_{t+1}{}^{i},b_{t+1}{}^{i},c_{t+1}{}^{i},d_{t+1}{}^{i}\right)}}\;*\;\sqrt{\frac{1+\vec{D_{t+1}{}^{i}}\left(a_{t}{}^{i},b_{t}{}^{i},c_{t}{}^{i},d_{t}{}^{i}\right)}{1+\vec{D_{t+1}{}^{i}}\left(a_{t+1}{}^{i},b_{t+1}{}^{i},c_{t+1}{}^{i},d_{t+1}{}^{i}\right)}},$$

where GTFP can be further decomposed into technical efficiency (EC) and technological progress (TC):

(6) $$\;\mathrm{EC}=\sqrt{\frac{1+\vec{D_{t+1}{}^{i}}\left(a_{t}{}^{i},b_{t}{}^{i},c_{t}{}^{i},d_{t}{}^{i}\right)}{1+\vec{D_{t}{}^{i}}\left(a_{t}{}^{i},b_{t}{}^{i},c_{t}{}^{i},d_{t}{}^{i}\right)}}\;*\;\sqrt{\frac{1+\vec{D_{t+1}{}^{i}}\left(a_{t+1}{}^{i},b_{t+1}{}^{i},c_{t+1}{}^{i},d_{t+1}{}^{i}\right)}{1+\vec{D_{t+1}{}^{i}}\left(a_{t+1}{}^{i},b_{t+1}{}^{i},c_{t+1}{}^{i},d_{t+1}{}^{i}\right)}},$$

(7) $$\;\mathrm{TC}=\frac{1+\vec{D_{t}{}^{i}}\left(a_{t}{}^{i},b_{t}{}^{i},c_{t}{}^{i},d_{t}{}^{i}\right)}{1+\vec{D_{t+1}{}^{i}}\left(a_{t+1}{}^{i},b_{t+1}{}^{i},c_{t+1}{}^{i},d_{t+1}{}^{i}\right)},$$

TC reflects the influence of external factors on DMUs, and EC reflects the utilization efficiency of resources at the current technological level. If the TC and EC indices are greater than 1, it indicates that DMU obtains technological progress, and the existing technology has a good utilization of resources. If the TC and EC indices are less than 1, it indicates that the external factors of DMU become worse and the utilization effect of existing technology on resources is poor.

To make further discussion in the following section (see Section 4.4), EC is composed of pure technical efficiency (PEC) and scale efficiency (SEC), the equations of which are as follows:

(8) $$\;\mathrm{SEC}=\sqrt{\frac{1+\vec{D_{t+1}{}^{i}}\left(a_{t}{}^{i},b_{t}{}^{i},c_{t}{}^{i},d_{t}{}^{i}\right)}{1+\vec{D_{t}{}^{i}}\left(a_{t}{}^{i},b_{t}{}^{i},c_{t}{}^{i},d_{t}{}^{i}\right)}},$$

(9) $$\;\mathrm{PEC}=\sqrt{\frac{1+\vec{D_{t+1}{}^{i}}\left(a_{t+1}{}^{i},b_{t+1}{}^{i},c_{t+1}{}^{i},d_{t+1}{}^{i}\right)}{1+\vec{D_{t+1}{}^{i}}\left(a_{t+1}{}^{i},b_{t+1}{}^{i},c_{t+1}{}^{i},d_{t+1}{}^{i}\right)}},$$

By solving linear programming problems for the four distance functions involved in Equation (5), we can finally obtain the constructions of the Malmquist productivity index.

#### 3.1.3. Regression Analysis

In order to investigate the impact of environmental regulation on the green total factor productivity, the following model is constructed:

(10) $$\;{\mathrm{GTFP}}_{it}=\theta_{0}+\theta_{1}lnEnv_{it}+\theta_{i}lnCon_{it}+\varepsilon_{it}+\mu_{it},$$

where i represents provinces and t represents years. Green total factor productivity (${\mathrm{GTFP}}_{it}$) is the explained variable. Environmental regulation ($Env_{it}$) is the main explanatory variable. $Con_{it}$ is the control variable, including foreign direct investment ($FDI_{it}$), government intervention ($Gov_{it}$), scale ($Sca_{it}$), and openness level ($Ope_{it}$).

### 3.2. Data Source and Variable Declaration

#### 3.2.1. Green Total Factor Productivity (GTFP)

This paper uses panel data of industrial sectors in nine provinces and two municipalities in the YREB to calculate green total factor productivity. Considering the availability of data, the research object of this paper is state-owned and above-scale industrial enterprises. Since the sixth National Environmental Protection Conference was held in 2007, on which the importance of environmental protection was emphasized, this paper began to study from 2008. Since this paper aims to comprehensively investigate YERB’s economic development from a viewpoint of green growth, i.e., green total factor productivity, labor input, capital investment, and energy consumption are involved in the inputs, indicators reflecting ecology and economy are taken as desirable outputs and sulfur dioxide emissions per unit of GDP are as undesirable outputs. Considering data availability, we use municipal household garbage harmless treatment rate as the indicator of ecology and comprehensive utilization rate of industrial waste as the economy. According to [21], the comprehensive utilization rate of industrial waste is due to the division of industrial solid waste by the quantity of industrial solid waste production. A perfect comprehensive utilization of industrial waste means that advanced facilities need to be invested, so that industrial waste can be handled in an environmentally friendly way, all of which are based on the high development level of the local economy. In other words, the high comprehensive utilization rate of industrial waste perfectly indicates the economy thus is chosen in this paper. The same logic applies to the municipal household garbage harmless treatment. That is, the higher the municipal household garbage harmless treatment rate, the better the condition of local ecology. Specifically, measurement indicators are shown in Table 1.

#### 3.2.2. Environmental Regulation

Environmental regulation has an important impact on green total factor productivity. At present, most scholars adopt the proportion of pollution control expenditure in GDP or the cost of handling unit pollutants to represent the environmental regulation stringency [21]. Considering the availability of the data, this paper adopts two indicators to represent the environmental regulation stringency: the operating cost of facilities that handle the industrial waste gas and water of the year and the number of the facilities [20,32,33,34].

#### 3.2.3. Control Variables

Foreign direct investment (FDI): It is generally believed that FDI affects green total factor productivity through capital formation, technology transfer, technology spillover, and environmental spillover, but the results are often regionally heterogeneous and related to the intensity of environmental regulation. This paper adopts the ratio of actual utilized FDI to GDP as an FDI variable [35,36].

Government intervention (Gov): Many studies show that the higher the degree of marketization, the better the green total factor productivity [37,38]. However, when the market fails, reasonable intervention from the government ensures the realization of economic goals. This paper uses the ratio of fiscal expenditure to GDP as a proxy of government intervention [39].

Industrial scale-up (Sca): the scale-up of industrial sectors can integrate resources, improve the overall technological level, and reduce resource waste, thus promoting the improvement of the green total factor productivity of enterprises. In this paper, the ratio of the GDP of medium and large enterprises to the whole industrial enterprises is used to measure the level of industrial scale [40].

Level of Opening to the outside world (Ope): Since the implementation of the reform and opening-up policy, China’s economy has developed rapidly, but the ecological environment has also deteriorated. At the present stage, scholars have different opinions on whether the improvement of the level of opening to the outside world hinders or promotes the green development of China’s economy. This paper adopts the viewpoint of Song et al. [41] to measure the level of opening up by employing the total amount of import and export as the level of opening to the outside world.

The data are from the China Statistical Yearbook, China Energy Statistical Yearbook and China Industrial Economic Statistical Yearbook of the National Bureau of Statistics of China (http://www.stats.gov.cn/20210631/, accessed on 17 November 2021).

## 4. Results and Discussion

### 4.1. GTFP Analysis

**Figure 2:**
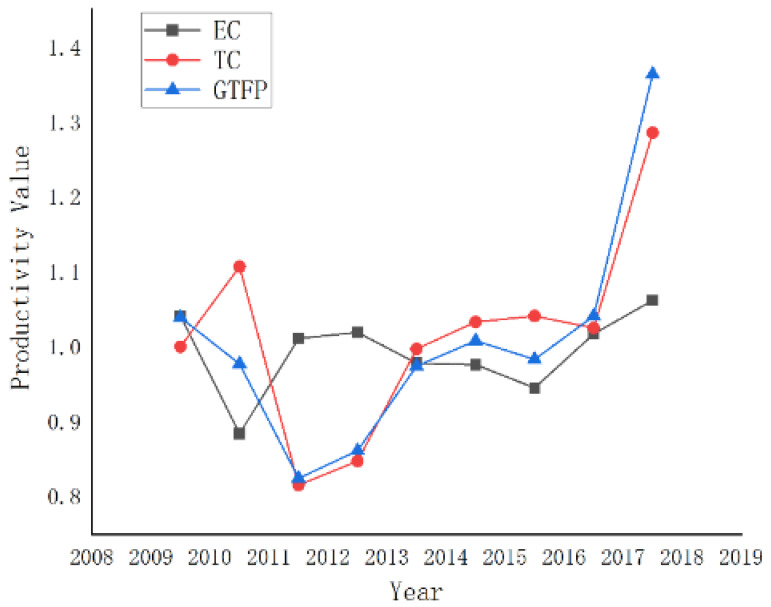
GTFP (Green Total Factor Productivity) and its decomposition (EC, technical efficiency and TC, technological progress) in the YREB (Yangtze River Economic Belt).

DEAP 2.1 software was used to measure the green total factor productivity of nine provinces and two municipalities in the YREB from 2009 to 2018, the measurement results are shown in Table 2 and Figure 2. As can be seen from Figure 2, the green total factor productivity in the YREB presents a trend of fluctuating growth, with an annual mean of 0.998, the average technical efficiency index is 0.99, and the technological progress index is 1.008.

Overall, the green total factor productivity in the YREB has improved but declined for several years before 2015. As can be seen from Figure 2, green total factor productivity in the YREB fluctuated greatly from 2009 to 2012, and both the technical efficiency index and technical progress index were less than 1 in some periods. This may be because the financial crisis in 2008 led to a prolonged period of economic stagnation in China, thus hindering the technological progress of various industries. Further, the plan to control greenhouse gas emissions was proposed for the first time during the 12th Five-Year Plan period, resulting in the insufficient utilization of resources for existing technologies. Therefore, the smooth development of the economy and environmental regulation policies has a significant impact on the improvement of green total factor productivity in the YREB [10,24].

The growing trend of technological progress index and technical efficiency index from 2009 to 2012 is similar to that of the green total factor productivity, with an overall upward trend and occasional downward trend. After 2012, environmental regulation policies were further intensified, as a result, various industries gradually realized the importance of technological progress and carried out transformation and upgrading strategies. As the main driving force of green total factor growth, technological progress promotes the development of productivity by upgrading production technology and improving the management level. After 2012, the intensified environmental regulation forced enterprises to upgrade technology, during this progress, green total factor productivity finally got improved. Therefore, the conclusion can be drawn that stringent environmental regulations force the upgrading of industrial technologies which are conducive to the green and sustainable development of the YREB [14,15].

### 4.2. Provincial Comparative Analysis

**Figure 3:**
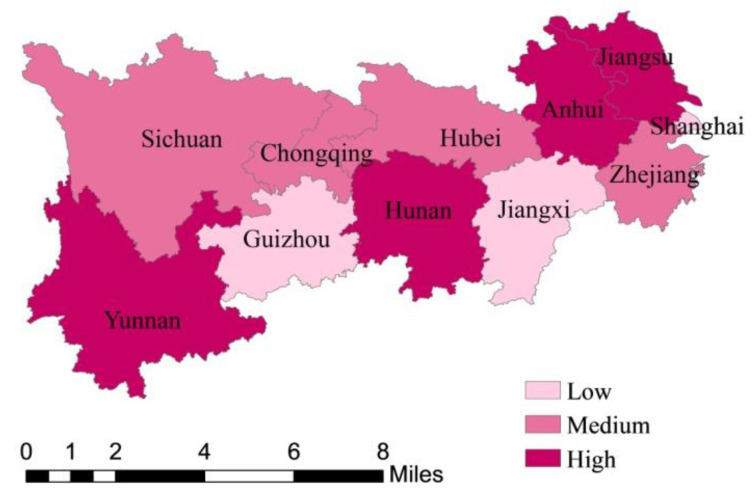
Spatial distribution of GTFP (Green Total Factor Productivity) in the YREB (Yangtze River Economic Belt) from 2009 to 2018.

**Figure 4:**
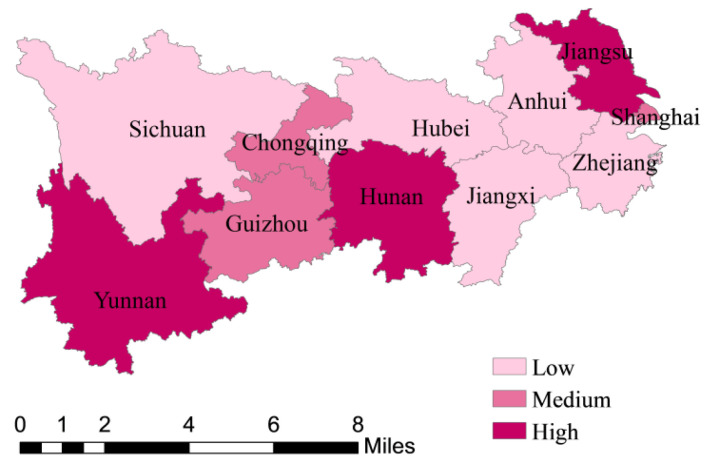
Same as Figure 3, but for the EC (technical efficiency) index.

**Figure 5:**
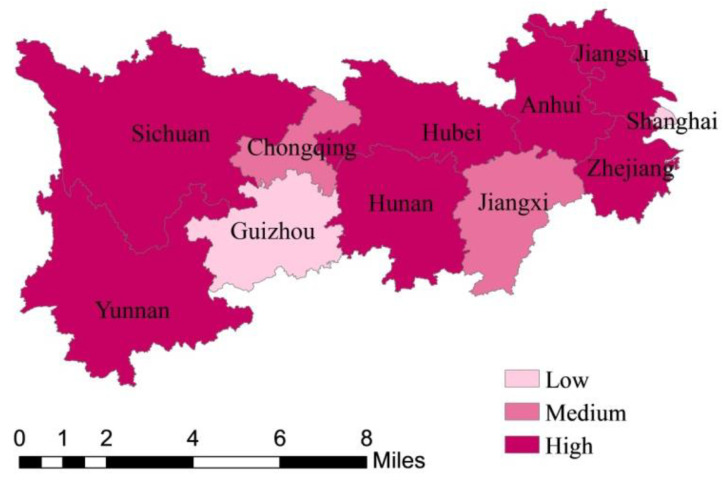
Same as Figure 3, but for the TC (technological progress) index.

To make a comparative analysis of GTFP and its decompositions, EC and TC, among provinces and municipalities more clarified, by setting indices below 0.92 as low, between 0.92 and 1 as medium, higher than 1 as high, Figure 3, Figure 4 and Figure 5 are drawn. As can be seen from Figure 3, the GTFP of which is based on the average levels from 2009 to 2018, that among the nine provinces and two municipalities in the YREB, the GTFP of Jiangsu, Hunan, Yunnan, and Anhui are all greater than 1, indicating relatively high green total factor productivity, while the rest provinces and municipalities are all less than 1, especially Jiangxi, Guizhou, and Shanghai, which are significantly lower than other provinces. Taken as a whole, the provinces and municipalities with higher green total factor productivity relatively coordinated the development of environmental governance and industrial economy from 2009 to 2018, this may be because the industrial economy is relatively developed, being in the former stage of technological development, due to which technological progress can significantly promote the improvement of resource utilization rate, or the environment itself is relatively good. By contrast, the provinces and municipalities with low green total factor productivity did not coordinate the development of environmental governance and industrial economy from 2009 to 2018.

It is worth mentioning that Shanghai, as the leader of research innovation and development in China, its TC index was lower than the EC index from 2009 to 2018. The reason may be that Shanghai’s industrial economy was in a transition period from 2009 to 2013: before 2013, the improvement of green total factor productivity was greatly influenced by technological progress; after 2013, with the industrial transferring and upgrading getting more completed and the free trade zone getting established, Shanghai’s economic green development presented significant advantages, and the impact of technological progress on the environment suggested a weakening trend. This is consistent with the study of Jiang et al. [42].

As can be seen from Figure 4 and Figure 5, the main influencing factor of GTFP in Zhejiang, Anhui, Hubei, Sichuan, and comes from technological progress, while Shanghai, Chongqing, and Guizhou are mainly affected by technological efficiency. In addition, Jiangsu, Hunan, and Yunnan are heavily influenced by both technological progress and efficiency.

As can be seen from Table 3, the green total factor productivity, the technological progress index, and the technological efficiency index of Jiangsu, Yunnan, and Hunan are all greater than 1 and there is little difference between the technological progress index and the technological efficiency index, which is manifested in the fact that their separate values of GTFPs are 1.323, 1.053, and 1.022; TC indices are 1.266, 1.034, and 1.006; and EC indices are 1.045, 1.018, and 1.015, respectively. It can also be seen that the green total factor productivity of Jiangsu, Yunnan, and Hunan are driven by both the technological progress and efficiency, the pattern of which is called two-way drive. In addition, TC indices of Zhejiang, Anhui, and Hubei are 1.02, 1.032, and 1.017, all greater than 1, but EC indices of these provinces are 0.933, 0.976, and 0.982, all less than 1, indicating low resource utilization rate and urgent need to improve technical efficiency in management and resource allocation. Moreover, TC indices of Shanghai, Jiangxi, Chongqing, and Guizhou are 0.919, 0.942, 0.957, and 0.916, and EC indices are 1, 0.963, 1, and 1, from which we can find that the technical efficiency index is higher than the technological progress index in these provinces, indicating that the innovation ability of these four provinces is not well transformed into production efficiency. In terms of provinces, the average TC index is 1.008, and the TC index of most provinces and municipalities is greater than 1, indicating that the overall technological level of the YREB makes progress, and all provinces and municipalities have high enough research and innovation ability. The mean EC index is 0.99, close to 1, but the EC index of almost half of the provinces is less than 1, indicating that the resource allocation efficiency of most provinces in the Yangtze Economic Belt needs to be improved. Based on the above results, hypothesis 1 is confirmed.

### 4.3. Impact of Environmental Regulations on GTFP

Due to the lack of data on the operation cost of facilities handling industrial waste gas and water after 2015, this paper only uses data from 2008 to 2014 to verify explanatory variables and explained variables. The classical OLS model is employed to test the relationship between GTFP and environmental regulation. To fix the potential heteroscedasticity problem, we first take the logarithm of the GTFP in Equation (10). Since VIF is less than 10, there is no multicollinearity between variables. After the Hausman test (*p*-value 0.01), the fixed-effect model is adopted in this paper. It can be seen from Table 4 that the regression coefficient of environmental regulation is positive, indicating that environmental regulation has a positive impact on the green total factor productivity of the YREB, but the impact is not significant. Due to the limitations of the data in this paper, the conclusions of this study may be biased. Combined with the research conclusions of Wang et al. [20] and Su et al. [23], it can be verified that the environmental regulation of the YREB locates on the right side of the U-shaped curve, and its promoting effect on the green total factor productivity is gradually weakening. Moreover, foreign direct investment has a significant positive impact on green total factor productivity, while government intervention has a significant negative impact. The regression coefficient of government intervention is negative, meaning that the government intervention is unreasonable and causes the loss of green total factor productivity in the YREB, which is in line with the conclusion of Lu et al. [35] The regression coefficient of FDI is positive, which suggests that foreign direct investment can promote green technology spillover and technology innovation in the YREB, thereby helping to improve regional green total factor productivity, which also confirms the study of Ayamba et al. [31]. Based on the above conclusions, hypothesis 2 is confirmed.

Due to omitted variables and reverse causality, the econometric model may present an endogeneity problem. On the one hand, there may be unobservable factors that can simultaneously influence FDI and GTFP; on the other hand, GTFP may, in turn, affect FDI, as places with better environmental regulation environments boast a higher level of GTFP thus higher FDI. To solve the bias caused by this problem, we select the excluded average FDI of other provinces and municipalities as the instrumental variable (IV) and perform the two-stage least squares (2SLS) regression, as shown in Table 5, which indicates that taking into account the probable endogeneity of the model, the regression result still supports the original finding that FDI has a positive impact on GTFP. In other words, the conclusion is robust. According to Ayamba et al. [31], for home countries, the inflow of FDI often means more advanced production technology and more stringent environmental standards. At the earlier stage of economic development, to stimulate the economy, local governments would allow FDI to flow inside without too many limitations; while, when the economy develops into a certain stage, the government will place more emphasis on local environmental situations and high environmental standards as well as introduce more advanced technology to improve the green total factor productivity.

### 4.4. Measurement Results of Environmental Governance Efficiency in the YREB

DEAP 2.1 software is used to calculate the input redundancy rate and output insufficiency rate of the environmental governance efficiency in the YREB. The input redundancy rate is the ratio of the corresponding input to the input slack variable, and the output insufficiency rate is the ratio of the corresponding output to the output slack variable. When the environmental governance efficiency is less than 1, the value of the relaxation variable explains the loss of environmental governance efficiency. The calculation results are shown in Table 6 and Table 7. As can be seen from Table 6, the environmental governance efficiency of Shanghai, Jiangxi, Chongqing, and Guizhou is 1, indicating that these four regions are effective in environmental governance. Therefore, Table 6 does not include these four regions. As can be seen from Table 6 and Table 7, taking the environmental governance process as a whole, industrial waste comprehensive utilization insufficient output rate is relatively low, except Hubei and Hunan, the rates of the other provinces are 0, indicating that the comprehensive utilization of the industrial waste in the YREB is high enough and the influence the industrial wastes have on the environment is not too much serious.

The EC of Anhui, Hubei, Hunan, Sichuan, and Yunnan is less than 1, so their environmental governance is ineffective, the reason may be that their scale does not match their input and output. The five provinces with ineffective environmental governance in Table 7 are classified into four categories by different reasons that lead to the environmental governance ineffectiveness (when the redundancy rate and insufficiency rate exceed 50%, it is assumed that this indicator is the main reason for ineffective invalid environmental governance). Jiangsu and Zhejiang are in the first category, with both of their pure technical efficiency values being 1 and scale efficiency value less than 1. Sichuan province belongs to the second category (with a relatively high input redundancy rate and a low output deficiency rate), and the third category includes Hunan and Anhui (with high undesired output rate and harmless treatment rate no more than 50%). Hubei and Yunnan are involved in the fourth category (with fairly high input redundancy and undesired outputs).

In terms of their own technical efficiency, Jiangsu and Zhejiang have no need to reduce input and increase output. Sichuan has a high input redundancy rate and a low output deficiency rate, indicating that it relies on a large amount of input to improve the environmental governance efficiency. Such effect may be significant in the short term, while in the long run, for enterprises, too much investment in the input is likely to result in a lack of innovation ability. The rate of harmless treatment in Anhui and Hunan is no more than 50%, but it is much closer; moreover, their undesired output is fairly high, indicating that the two provinces lag behind in environmental regulation equipment and technology, remain in the middle and low end of the industrial chain, and lack the ability of innovation and research and development (R&D). The environmental governance ineffectiveness in Hubei and Yunnan lies not only in the redundancy of input factors but also in the excessive undesired output, suggesting that their environmental governance is in a state of extensive development with high input and high output [25].

## 5. Conclusions and Recommendations

This paper selected nine provinces and two municipalities in the YREB from 2009 to 2018 as the research object, employs the SBM model to measure green total factor productivity, and analyzes the green total factor productivity of each province and its change. At the same time, areas with ineffective environmental governance are analyzed and the main reason for such ineffectiveness is found. Conclusions are drawn as follows: Firstly, the overall environmental governance in the YREB was improved and began developing in an increasingly effective manner during the studied period. From 2009 to 2018, Shanghai, Chongqing, Guizhou, and Jiangxi were all effective in environmental governance. Meanwhile, although the rest of the YREB were ineffective, their environmental governance capacity improved. Secondly, from the perspective of the contribution degree, technological progress contributes more to the overall GTFP of YREB with its mean value of 0.923, higher than the mean value of the technical efficiency—0.840 (see the lase line of Table 3). Furthermore, when it comes to the decomposition of the technical efficiency (Table 5), the scale efficiency (SEC) contributes more than the pure technical efficiency (PEC), whose means are 0.923 and 0.840, respectively. That is, the pure technical efficiency has the weakest impact on GTFP. Lastly, in terms of the environmental governance process, redundant input, excessive undesirable output, and low harmless treatment rate of municipal solid waste are the reasons for the ineffectiveness of environmental governance in the YREB, this is because municipal solid waste is the main product of household garbage, and please remember that the evaluation indices of the desired outputs of GTFP in this paper include municipal household garbage harmless treatment rate (see details in Table 1). Based on the above conclusions, this paper puts forward the following recommendations.

Firstly, improve the efficiency of resource allocation and save input factors. From 2009 to 2018, the efficiency of environmental governance in the YREB showed a trend of fluctuated rising. Technological progress was the main driving force of green total factor productivity, and the driving effect of technological efficiency was not obvious. Since technological progress requires continuous input factors to make the production technology keep improving, while technical efficiency is generally used to explain the efficiency of resource allocation and the saving of input factors, on the premise of continuous innovation of production technology, to eliminate the negative impact of technological efficiency on GTFP, the YREB should focus on improving resource allocation efficiency and saving input factors. Government departments should establish a reasonable resource price system and provide critical support to resource-conserving and resource-comprehensively utilizing projects and encourage industrial enterprises to invest in research and development and apply resource-saving technologies.

Secondly, make full consideration of strengths and weaknesses of different provinces and municipalities and implement regionally appropriate environmental governance policies to develop the economy. Since the environmental governance outcomes vary from province to province, environmental regulation policies should also take both the regional economy and environmental characteristics into account to ensure healthy economic development. Specifically, design reasonable policies to effectively improve the environment quality, for example, in areas where the technological progress index is lower than 1, enterprises should be encouraged by the government to step up efforts to promote innovation, remove the threshold effect and accelerate the transformation of the digitizing and informatizing. Increase the investment to the infrastructure and environmental governance and improve the technological transformation rate due to the fact that the technological efficiency indices of the most studied provinces are low. Overall, innovation outcomes should be transformed into the powerful engine of enterprises.

Thirdly, establish targeted plans by the different reasons for ineffective environmental governance. According to four kinds of environmental governance ineffectiveness proposed in Section 4.4, recommendations are as follows: (i) For ineffectiveness caused by low scale efficiency, Zhejiang and Jiangsu should expand the industrial scale and improve the production efficiency, while increasing input or reducing output is not necessary. (ii) For ineffectiveness caused by redundant input, Sichuan should guide enterprises to transform into energy-saving and environment-friendly patterns and cut the investment in environmental governance and human resource. Meanwhile, investment in advanced technology introduction and senior environmental talents should still be encouraged. (iii) For ineffectiveness caused by excessive undesired output and insufficient comprehensive utilization of industrial waste, Anhui and Hunan need to improve the environmental financing mechanism to speed up the industrial transformation to green development and raise funds for the research of energy efficiency utilization and improvement. (iv). For ineffectiveness simultaneously caused by excessive input and undesirable output, Hubei and Yunnan should invest more in technology improvement and research and development (R&D), on the premise of keeping the current resource input and allocation balanced. This is because the increasing environmental governance investment in these provinces did not work out as expected, which means such investment is simply a substantial waste of resources and should be reallocated.

There are some limitations that require further research. From the perspective of industry, this paper mainly discusses the environmental governance in the nine provinces and two municipalities of the YREB, however, industry alone boasts many niche segments such as manufacturing, mining, etc., and each sector has its own characteristics, thus different impacts on the environment. Therefore, future research should focus on the specific characteristic of different industrial sectors and explore the environmental governance of the YREB from different angles. In addition, although industry is the pillar of the national economy and it is of great significance to discuss the change of green total factor productivity of industry, the growth of green total factor productivity of agriculture, service industry, and other industries should also be emphasized as they are essential to the green development of the economy, which is ignored in this paper. In future research, the green development of various industries and the influence of their interaction on the green development of the economy should be comprehensively studied.

## Figures and Tables

**Table 1:** GTFP evaluation indices.

Vector	Serial Number	Index	Measurement	Reference
Inputs	A1	Labor input	Total employment of industrial enterprise	[23]
	A2	Capital investment	Average annual balance of net fixed assets of Industrial enterprise	[29]
	A3	Energy Consumption	Total energy consumption of Industrial enterprise	[21]
Desirable outputs	B1	Ecology	Municipal household garbage harmless treatment rate	[25]
	B2	Economy	Comprehensive utilization rate of industrial waste	[30]
Undesirable outputs	C1	Pollution	Sulfur dioxide emissions per unit of GDP	[31]

Note: All measurements (i.e., GDP, employment, output) are provincial scale. The data are from China Statistical Yearbook and Provincial Statistical Yearbook.

**Table 2:** GTFP and its decomposition in the YREB from 2009 to 2018.

Year	EC	TC	GTFP
2009~2010	1.04	0.999	1.038
2010~2011	0.883	1.106	0.976
2011~2012	1.01	0.814	0.823
2012~2013	1.018	0.846	0.86
2013~2014	0.977	0.996	0.973
2014~2015	0.975	1.032	1.007
2015~2016	0.944	1.04	0.982
2016~2017	1.016	1.024	1.04
2017~2018	1.061	1.285	1.363
Mean	0.99	1.008	0.998

**Table 3:** GTFP of provinces and municipalities in the YREB and its decomposition.

	EC	TC	GTFP
Shanghai	1	0.919	0.919
Jiangsu	1.045	1.266	1.323
Zhejiang	0.933	1.02	0.952
Anhui	0.976	1.032	1.008
Hubei	0.982	1.017	0.999
Hunan	1.015	1.006	1.022
Jiangxi	0.963	0.942	0.908
Chongqing	1	0.957	0.957
Sichuan	0.964	1.014	0.977
Guizhou	1	0.916	0.916
Yunnan	1.018	1.034	1.053
mean	0.99	1.008	0.998

Note: EC: technical efficiency; TC: technological progress; GTFP: green total factor progress.

**Table 4:** Regression results.

Variable	Coefficient	*t* Value	*p*-Value
Env	0.2262853	1.66	0.104
FDI	0.2941534	2.48	0.017
Sca	0.7291552	1.14	0.261
Gov	−1.148243	−4.00	0.000
Ope	0.0559033	0.34	0.735

Note: Env: environmental regulation; FDI: foreign direct investment; Sca: industrial scale-up; Gov: government intervention; Ope: level of opening to the outside world.

**Table 5:** The 2SLS estimation of the instrumental variable.

	First Stage LS	Second Stage LS
IV	0.3657 * (1.60)	
FDI		0.0254 * (1.64)

Note: LS: least squares regression; IV: instrumental variable; FDI: foreign direct investment. * mean that the variable coefficient has passed the significance test at the level of 10%, the corresponding *t* values are in parentheses.

**Table 6:** Environmental governance efficiency of the YREB (Yangtze River Economic Belt).

	EC	PEC	SEC
Shanghai	1.000	1.000	1.000
Jiangsu	0.676	1.000	0.676
Zhejiang	0.640	1.000	0.640
Anhui	0.643	0.644	0.998
Hubei	0.535	0.566	0.946
Hunan	0.651	0.684	0.952
Jiangxi	1.000	1.000	1.000
Chongqing	1.000	1.000	1.000
Sichuan	0.532	0.549	0.970
Guizhou	1.000	1.000	1.000
Yunnan	0.767	0.794	0.966
mean	0.768	0.840	0.923

Note: EC: technical efficiency, also known as comprehensive efficiency, PEC: pure technical efficiency, SEC: scale efficiency, EC = PEC ∗ SEC.

**Table 7:** Input-output analysis of environmental governance efficiency in the YREB (Yangtze River Economic Belt).

	Input Redundancy Rate (%)			Under-Output Rate (%)		
	Input 1	Input 2	Input 3	Output 1	Output 2	Output 3
Jiangsu	0.00	0.00	0.00	0.00	0.00	0.00
Zhejiang	0.00	0.00	0.00	0.00	0.00	0.00
Anhui	35.77	35.63	35.63	48.65	0.00	61.57
Hubei	43.45	74.64	43.44	71.67	5.72	142.02
Hunan	31.61	46.90	31.61	43.65	5.08	88.71
Sichuan	45.13	45.13	64.56	6.54	0.00	16.33
Yunnan	20.61	20.61	54.67	3.86	0.00	176.54

## Data Availability

The datasets generated during and/or analyzed during the current study are available in the State Statistical Bureau, China Statistical Yearbook 2008–2018 repository https://data.stats.gov.cn/ (accessed on 6 January 2021).
